# Public’s Attitudes Toward Health Information on Twitter: A Cross-sectional Survey Based on the Saudi Population

**DOI:** 10.7759/cureus.5863

**Published:** 2019-10-08

**Authors:** Shareefah A Alassiri, Areej S Alowfi

**Affiliations:** 1 Administration of Public Health, Ministry of Health, Jeddah, SAU; 2 Family Medicine, King Saud Bin Abdulaziz University for Health Sciences / King Abdulaziz Medical City, Ministry of National Guard Health Affairs, Jeddah, SAU

**Keywords:** twitter, health information, saudi arabia

## Abstract

Health information dissemination through social media networks has transformed the process of communication between health practitioners, patients, and the public. Twitter is one of the most commonly used social media networks in Saudi Arabia for sharing health information. However, the reliability of health information on Twitter has been questioned by some skeptics, thereby placing the public at a significant health risk. This study was conducted to assess the attitudes of the general population of Saudi Arabia towards health information sought from Twitter. Of the 384 total respondents, 199 (51.8%) considered using Twitter as a source of health information as a positive experience due to its ease of use and the accessibility of information (131, 66.0%). The study found that respondents have great concern about nutrition/weight loss (229, 59.6%), healthy lifestyle (225, 58.6%), and getting a better understanding of health care misconceptions (168, 43%). Most of the respondents (167, 43.5%) were satisfied using Twitter as a source of health information. However, a few respondents experienced harm to their health as a result of false medical advice (2, 0.5%) and false health information (2, 0.5%) they found on Twitter. This study concludes that Twitter is mainly useful in obtaining health knowledge for maintaining good health, preventing illness, and curing illnesses or diseases. However, the public must take extra caution when obtaining health information from Twitter. It is essential for Twitter users seeking medical information to also seek professional medical advice or consultation, as necessary, to prevent a significant health risk.

## Introduction

The dissemination of health information during this digital generation has emerged in various ways to convey messages to the target population or audience. Social media network sites and the availability of web-based platforms that facilitate information sharing in the form of user-generated content have indeed transformed the process of communication.

According to the Internet Live Stats website, 40% of today’s world population has an Internet connection [1]. In addition, the unprecedented number of people using smartphones for social and digital media provides an opportunity for health practitioners and institutions to communicate and disseminate health information among individuals and to the public concerning health care issues [2].

A survey of 111 patients from an outpatient family practice clinic in southern Utah, United States, showed that 56% wished that their physicians should use social networking platforms for health information sharing, answering general questions, setting appointments and reminders, notifying prescriptions, and reporting diagnostic test results [3].

Physicians are currently exploring social media network platforms as a way to seamlessly share health information with relevant health care stakeholders. For instance, social networks are being utilized to seek and share health information, to be a source of communication with trainees and colleagues, to market physicians’ practices, to disseminate research, and to spread health recommendations. Moreover, direct interaction with patients has been growing through the use of social media networks [4-6]. However, skeptics may argue about the efficiency and safety of using Twitter as a social media networking tool in gaining health information for the public and for medical and allied health professionals [7].

Therefore, this study aims to assess the attitudes of the general population of Saudi Arabia towards seeking health information through Twitter.

## Materials and methods

This is a cross-sectional study conducted among Twitter users in Saudi Arabia to determine their attitudes towards seeking health information on Twitter as a medium of information. Inclusion criteria in the selection of the respondents were: Twitter users living in Saudi Arabia (i.e., Saudi and non-Saudi nationals), at least 18 years of age, and those who used Twitter to search and/or seek health information. The minimum sample size was estimated to be 385 using the online Raosoft Inc. sample size calculator (WA, US) [8] based on the total number of Twitter users in Saudi Arabia (i.e., approximately 5 million) [9] and with an acceptable margin of error of 5% and a confidence level of 95%.

The researchers designed a self-administered survey questionnaire in the English and Arabic languages. Two family medicine consultants who are native Arabic speakers validated the questionnaire for the construct, content, and face validity. The survey questionnaire consisted of four parts: questions pertaining to the respondents’ sociodemographic data, respondents’ characteristics as a Twitter user, health-related benefits and harms from seeking health information from Twitter, and reasons for using Twitter to search for health information. A pilot test was conducted twice (i.e., an initial pilot survey and a follow-up survey after one week) to 20 randomly selected Twitter users who volunteered to test for internal consistency (Cronbach’s alpha 0.89) and the test-retest reliability (alpha 0.80) of the questionnaire. The final survey questionnaire was distributed using the exponential non-discriminative snowball sampling technique. A survey link posted as a tweet by the researchers and shared among Twitter users with different traits or demographic characteristics as strata to reach out to a greater number of the Saudi population. The survey link directed respondents towards the SurveyMonkey platform with an option to change the language based on the preferred language of the respondents. Respondents consented to participate by submitting the completed survey questionnaire. The survey was conducted from June 7, 2016, to June 30, 2016. All collected data about the respondents were stringently maintained to be anonymous and confidential throughout the study.

All collected data were analyzed using the Statistical Package for the Social Sciences software, version 21 (SPSS v.21; IBM Corp, Armonk, NY, US). Frequency and percentage were used for describing categorical data. The chi-square test (χ2) was used to test for the association between categorical variables. A p-value of ≤ 0.05 was considered significant.

Permission to conduct the study was obtained from the Department of Research and Ethics Committee and the Program Director of Family Medicine of Prince Sultan Military Medical Center, Riyadh, Saudi Arabia, before commencing the study, with the supervision of the Joint Program of Family and Community Medicine.

## Results

Of the 401 respondents who participated in the survey, 384 (95.76%) fulfilled the inclusion criteria and were included in the final analysis. Most respondents were aged 18 to 35 years (309, 80.5%) and most were male (242, 63.0%). There were 360 (93.8%) who were Saudi nationals and 24 (6.2%) who were non-Saudi nationals. There were 198 (51.6%) from the region of Makkah, and there were 234 (60.9%) who had tertiary education. The majority, 129 (33.6%), worked in the medical field, followed by 78 (20.3%) students. There were 224 (58.4%) respondents who reported a monthly income of more than 12,000 Saudi Riyals (i.e., US$ 3,200 or €2,925). Table 1 presents the complete socio-demographic characteristics of the respondents.

**Table 1:** Socio-demographic characteristics of the respondents

Socio-demographic characteristics		Frequency (n)	Percentage (%)
Age	18–25	142	37.0
	26–35	167	43.5
	36–45	47	12.2
	> 45	28	7.3
Gender	Male	242	63.0
	Female	142	37.0
Nationality	Saudi	360	93.8
	Non-Saudi	24	6.2
Marital Status	Single	200	52.1
	Married	178	46.4
	Divorced	6	1.5
	Widow	0	0.0
Region	Makkah	198	51.6
	Riyadh	87	22.7
	Assir	45	11.7
	Madinah	24	6.2
	Jazan	5	1.3
	Eastern Region	5	1.3
	Others	20	5.2
Educational Attainment	Intermediate	8	2.1
	Secondary	49	12.8
	University	234	60.9
	Diploma	22	5.7
	Masters	47	12.2
	Doctorate	24	6.3
Field of Occupation	Unemployed	52	13.5
	Student	78	20.3
	Medical	129	33.6
	Educational	38	9.9
	Engineering	7	1.8
	Administration	35	9.1
	Business	14	3.6
	Retired	8	2.1
	Other	23	6.1
Monthly Income (Saudi Riyals)	≤ 1000	4	1.0
	1001–3000	28	7.3
	3001–6000	28	7.3
	6001–9000	37	9.6
	9001–12,000	63	16.4
	> 12,000	224	58.4

Most of the respondents have a Twitter user for one to five years (283, 73.6%). The majority of respondents have a positive response (199, 51.8%) to using Twitter as a source of health information, compared to a negative response (185, 48.2%). Moreover, most of the respondents (131, 66.0%) preferred Twitter due to its ease of use and access. Respondents have mostly sought information about physicians (139, 36.2%) and government health institutions (105, 27.3%). Table 2 shows the characteristics of respondents who use Twitter to obtain health information.

**Table 2:** Characteristics of respondents who use Twitter to obtain health information

Characteristics of respondents who use Twitter	Frequency (n)	Percentage (%)
Duration of Twitter use		
< 1 year	29	7.6
1–3 years	106	27.5
4–5 years	177	46.1
> 5 years	72	18.8
Preference of Twitter use for health information		
Positive	199	51.8
Negative	185	48.2
Reason for preference		
Ease of use and access	131	66.0
Comprehensive information	32	16.0
Validity of information	10	5.0
Low cost	15	7.5
Other	11	5.5
Seeking health information through accounts belonging to:		
Governmental health institutions	105	27.3
Private health institutions	15	3.9
Medical organizations	43	11.2
Health campaigns	27	7.1
Particular physicians	139	36.2
Particular specialty search	19	4.9
Other	36	9.4

As shown in Table 3, most respondents said that using Twitter to obtain health information will provide a better understanding of misconceptions (168, 43%). They also used Twitter mainly to search for general knowledge (256, 66.7%), disease prevention (170, 44.3%), and cures to illness (154, 40.1%). Of the searched health specialties on Twitter, nutrition (229, 59.6%) and healthy lifestyle (225, 58.6%) were the most searched items. Reasons for seeking health information in a particular medical specialty are shown in Table 4.

**Table 3:** Benefits and harms of obtaining health information through Twitter

Items	Frequency (n)	Percentage (%)
Benefits from Twitter use		
Better diet	94	24.5
Abstinence from smoking	4	1.0
Weight loss	39	10.2
Better health care for my family	30	7.8
Better understanding of misconceptions	168	43.8
Other	49	12.7
Level of satisfaction of obtaining health information through Twitter		
Very satisfactory	25	6.5
Satisfactory	167	43.5
Neutral	161	41.9
Unsatisfactory	31	8.1
Distribution of respondents who experienced harm caused by false health information on Twitter		
A harm experience	2	0.5
No harm experience	312	81.3
Don’t know	70	18.2
The harm caused by the application of information obtained from Twitter		
Pain in teeth implant	1	0.05
Severe dizziness	1	0.05
Visited the hospital due to harm from the application of information obtained from Twitter		
Yes	2	0.5
Never	382	99.5

**Table 4:** Reasons for searching health information on Twitter

Items	Frequency (n)	Percentage (%)
Reasons for Twitter search		
Disease prevention	170	44.3
Search of a cure for my illness	154	40.1
Epidemic outbreaks	109	28.4
General knowledge	256	66.7
Twitter search according to health specialties		
Nutrition/weight loss	229	59.6
Healthy lifestyle	225	58.6
Family medicine	125	32.6
Cosmetics	90	23.4
Cancer and its prevention	75	19.5
Dermatology	61	15.9
First aid	61	15.9
Dental	59	15.4
Internal medicine	56	14.6
Pediatrics	44	11.5
Ob-Gyne	43	11.2
Other	43	11.2
Infectious diseases	39	10.2
Heart diseases	36	9.4
Sexually transmitted diseases	28	7.3
Pulmonology	26	6.8

Table 5 shows that the age group of 18-25 (144, 37.5%) and 26-35 (166, 43.2%) and the benefits gained from seeking health information on Twitter were all statistically significant (p = 0.01). Lastly, professionals in the medical field (129, 33.6%) have experienced favorable benefits from Twitter as compared to other occupations. The correlation between the benefits gained from Twitter use and the respondents’ occupation show statistical significance (p<0.001) (Table 6).

**Table 5:** Association between the benefits of Twitter use and age group

Benefits from Twitter use	Age Group				P-Value
	18–25	26–35	36–45	> 45	
Better diet	45	33	8	8	0.01
Abstinence from smoking	0	4	0	0	
Weight loss	13	18	7	1	
Better health care for my family	6	17	7	0	
Better understanding of misconceptions	71	66	18	13	
Other	9	28	7	6	

**Table 6:** Relationship between the benefits of Twitter use and the respondents’ occupational field

Occupational field	Benefits from Twitter use						P-Value
	Better diet	Abstinence from smoking	Weight loss	Better health care for family	Better understanding of misconceptions	Other	
Unemployed	14	0	2	2	31	3	< 0.001
Student	23	0	0	2	8	6	
Medical	24	2	13	15	47	28	
Educational	10	2	5	1	20	0	
Engineering	1	0	3	0	0	3	
Administration	9	0	4	7	14	1	
Business	4	0	0	2	8	0	
Retired	2	0	4	0	3	3	
Other	7	0	4	1	6	5	

## Discussion

As a growing proportion of individuals seek health information online and through social media platforms, communication about health has become increasingly interactive and dynamic. Twitter is one of the most commonly used social media platforms due to its popularity among the global audience, easy access to information, and a medium that quickly spreads information [10]. The results of this study show an increase in the number of Twitter users in Saudi Arabia who seek health information, as more than half (131, 51.8%) of the respondents preferred Twitter as a source of health information due to its ease of use and accessibility. With this widespread popularity of Twitter come new challenges in the aspects of communication and learning about health information across users of different age groups [11].

Nowadays, the dissemination of health information on diseases and treatment is almost entirely through social media. Twitter, for instance, has been shown to be an effective platform in the dissemination of information [12]. In the current study, more than half of the respondents (199, 51.8%) expressed a positive attitude towards the use of Twitter to search for health-related information. As evidence, 256 (66.7%) of the respondents used Twitter to obtain general knowledge, perhaps because most of the respondents of this study were from the medical field.

Chew et al. conducted a study on the dissemination of health information through Twitter. They reviewed Twitter status on antibiotics updates, determined the overarching categories, and explored evidence of misunderstanding or misuse of antibiotics. The study concluded that social media offered a means of health information sharing. However, further research was suggested to determine the misunderstanding or misuse of antibiotics among Twitter users. Thus, to disseminate valid information, promote positive behavior change among Twitter users, and explore how Twitter can be used as a tool to gather real-time health data [13]. This result is similar to what the current study results imply. Although most of the respondents expressed a positive attitude (199, 51.8%) towards Twitter use particularly in the aspect of nutrition (229, 59.6%) and healthy lifestyles (225, 58.6%), this study found that most of the respondents (281, 73.2%) doubted the credibility of the information available on Twitter.

In the study of Brady et al., the use of Twitter had demonstrated a measurable impact on strengthening the collaboration among physicians. Enriching their ability to communicate and share ideas with one another effectively and to bring issues need to be discussed [14]. A recent study verified over 2,000 doctors on Twitter based on their National Provider Identifier. These doctors have at least 300 followers each and tweet more than once per day [15]. The results of the current study showed that more than one-third of respondents (139, 36.2%) sought health information through accounts belonging to physicians.

The accuracy of health information is one of the major concerns for Twitter users. Chretien et al. studied the prevalence of misinformation in tweets about health care, and the results revealed that about 20% contained inaccurate information. The study also stressed the lack of a systematic process for misinformation checking on Twitter [16]. Choo et al. also agreed that it is quite easy for a non-expert to present their opinions as facts and appear to be credible as an unbiased medical professional [17].

The findings of the current study were aligned with those of the studies mentioned above. When the respondents asked if the health information they obtained from Twitter had met their needs, half of the respondents were satisfied (very satisfactory: 25, 41.9%; satisfactory: 167, 43.5%) and a few were unsatisfied (31, 8.1%). Two respondents (0.5%) claimed that they were harmed due to following the false health advice they received on Twitter. Another two respondents (0.5%) claimed that they needed to visit a hospital as a result of following the false health information from Twitter (Table 3). Therefore, these findings must spur Twitter users to validate the credibility of health information they get from Twitter or, better yet, to seek professional medical advice to avoid similar circumstances.

The data analyzed in this study were based on the total number of respondents who voluntarily participated and completed the survey questionnaire. Since the majority of the respondents who participated were from medical backgrounds while there was a relatively low sample size in other occupational fields, the multiple variable analysis would be at risk of bias from multiple comparisons. However, based on the results of the study, we recommend conducting further research focusing on a non-medical background population to improve the generalization of this study’s results. The reliability of health information on Twitter noticed by the respondents was based on their own understanding. Researchers did not assess the content of health information and the credibility of sources.

## Conclusions

Searching for health information through Twitter is increasing among the population of Saudi Arabia. The health information on Twitter is somewhat questionable for some of the respondents, and some had experienced harm due to false health advice and information. On this matter, the public must be extra cautious of all health information obtained from Twitter. Moreover, it is essential for Twitter users to seek professional medical advice or consultation, as necessary, to prevent a significant health risk.
